# Impact of neurofibromatosis type 1 with plexiform neurofibromas on the health-related quality of life and work productivity of adult patients and caregivers in the UK: a cross-sectional survey

**DOI:** 10.1186/s12883-023-03429-7

**Published:** 2023-11-23

**Authors:** Hyun Kyoo Yoo, Alex Porteous, Alvin Ng, Keval Haria, Annabel Griffiths, Andrew Lloyd, Xiaoqin Yang, Gbenga Kazeem, Volkan Barut

**Affiliations:** 1 Alexion, AstraZeneca Rare Disease, Cambridge, UK; 2 Costello Medical, London, UK; 3 Costello Medical, Singapore, Singapore; 4 Costello Medical, Cambridge, UK; 5 Acaster Lloyd Consulting, London, UK; 6 Merck & Co., Inc, Rahway, NJ USA; 7 AstraZeneca, Cambridge, UK

**Keywords:** Neurofibromatosis, Plexiform neurofibromas, Quality of life, Utility, EQ-5D, Caregivers

## Abstract

**Background:**

Plexiform neurofibromas (PN) are complex, benign nerve-sheath tumours that occur in 30–50% of patients with neurofibromatosis type 1 (NF1), a rare, genetic disorder. PN are associated with substantial, heterogeneous morbidities that impact health-related quality of life (HRQoL), including affecting motor function and causing pain, though HRQoL and work productivity data are scarce. This UK cross-sectional study explored HRQoL and work productivity in adult patients with NF1 PN and caregivers of paediatric patients.

**Methods:**

Adult patients and caregivers of paediatric patients self-enrolled in an online survey (March–April 2021). Outcomes included EQ-5D-5L, PROMIS® GH and INF1-QOL (adult patients only), and EQ-5D-5L, CarerQol and WPAI (caregivers only). Utilities were estimated from EQ-5D-5L responses using the UK crosswalk value set. Linear regression models explored univariable associations between adult patient characteristics and HRQoL.

**Results:**

Mean (± standard deviation) EQ-5D utility in adult patients with NF1 PN was 0.65 (± 0.29; *n* = 35; age-/sex-matched norm: 0.89 [± 0.04]). Moderate–extreme pain/discomfort and anxiety/depression were reported by 14/35 (40.0%) and 18/35 (51.4%) patients, respectively. Mean PROMIS® GH physical and mental health scores were 43.6 (± 9.19) and 41.7 (± 11.5; *n* = 35; matched norm: 50.0 [± 10.0]). Mean INF1-QOL score was 11.03 (± 6.02; *n* = 33). Chronic itching, at least one symptom, at least one comorbidity, PN location at extremities (arms/legs) and pain were associated with worse HRQoL scores.

Mean caregiver EQ-5D utility was 0.72 (± 0.24; *n* = 8; age-/sex-matched norm: 0.88 [± 0.03]). Moderate pain/discomfort and moderate–severe anxiety/depression were reported by 4/8 (50.0%) and 2/8 (25.0%) caregivers, respectively. Mean CarerQol score was 69.3 (± 13.9; *n* = 8). Mean WPAI regular activity productivity loss was 36.3% (± 31.6%; *n* = 8).

**Conclusions:**

NF1 PN worsens adult patient and caregiver HRQoL compared to the general population, notably affecting pain and discomfort, anxiety and depression and caregiver productivity.

**Supplementary Information:**

The online version contains supplementary material available at 10.1186/s12883-023-03429-7.

## Background

Neurofibromatosis type 1 (NF1) is a progressive, genetic disorder affecting approximately 1 in 3000 children and adults in European populations [1, 2]. A common feature of NF1, reported in 30–50% of patients [3, 4], is the presence of plexiform neurofibromas (PN). PN are benign but often progressive tumours arising from nerves [5], which can occur anywhere on the body. They can cause substantial, heterogeneous morbidities, often due to their size, location and invasiveness [6], including pain, disfigurement and motor deficits, as well as potentially life-threatening complications such as airway obstruction [6–10].

Data on the impact of NF1 (particularly with PN) on the health-related quality of life (HRQoL) and work productivity of patients or their caregivers, including health utilities, remain scarce. A small number of studies have suggested that patients with NF1 have poorer HRQoL compared with the general population, with emotional wellbeing, social functioning, mental health, appearance, sleep, pain and daily activities affected [7–15]. Negative emotional, social and productivity impacts have also been observed in caregivers of patients with NF1 PN [15, 16].

We report results from an exploratory study conducted to quantitatively assess the impact of NF1 PN on HRQoL and caregiver work productivity, and estimate utilities for adult patients with NF1 PN and caregivers of paediatric patients. We also report on the relationship between patient characteristics and HRQoL.

## Methods

This exploratory, cross-sectional, non-interventional study consisted of two anonymous surveys directed at adult patients with NF1 PN and caregivers of paediatric patients with NF1 PN, respectively, in March–April 2021. The study followed a pre-specified protocol including a statistical analysis plan. Ethical approval was obtained from London – West London & Gene Therapy Advisory Committee Research Ethics Committee.

### Patients and procedures

Eligible patients and caregivers were > 18 years of age, able to read and write in English and residents of the United Kingdom (UK). Fulfilment of eligibility criteria was self-reported to maintain anonymity. Patients reported having a clinical diagnosis of NF1 with at least one PN, and were receiving established clinical management without mitogen-activated protein kinase kinase (MEK) inhibitors (including selumetinib, binimetinib, cobimetinib, mirdametinib and trametinib) which were being assessed as treatments for NF1 PN. Patients who were pregnant were excluded from the study to align with eligibility criteria of clinical trials for patients with NF1 PN. In order to preserve participant anonymity, participants gave their consent via a tick box at the start of the survey.

The survey was distributed via SurveyMonkey (SurveyMonkey Inc., San Mateo, California, USA; http://www.surveymonkey.com).

### Patient organisation involvement

Nerve Tumours UK (NTUK), a patient organisation, advised on the feasibility of recruitment, measures used in the survey, study protocol, participant information forms and the Integrated Research Application System (IRAS) application form. NTUK reviewed the survey and distributed the recruitment advertisement (containing the weblink to the survey) via their website, email newsletters and social media platforms.

### Outcomes

The choice of patient and caregiver HRQoL and work productivity measures used in this study was informed by a targeted literature review of previous studies in NF1 and related conditions. Patients completed generic and disease-specific HRQoL measures previously used and/or validated in NF1, including the EQ-5D-5L, the Patient-Reported Outcomes Measurement Information System® Global Health (PROMIS® GH) and the Impact of NF1 on Quality of Life (INF1-QOL) measure. Caregivers completed the generic HRQoL measure EQ-5D-5L, as well as quality of life (QoL) and work productivity measures related to informal caregiving, including the Care-Related Quality of Life (CarerQol) measure and the Work Productivity and Activity Impairment questionnaire as adapted for caregiving (WPAI:CG). Table 1 further describes each measure and its relevance to patients with NF1 and caregivers.

**Table 1:** Summary of HRQoL and work productivity measures completed by patients and caregivers in the survey

Measure	Type	Justification for use in the survey	Scale
**Adult patients**			
EQ-5D-5L	Generic (HRQoL)	Previously used in NF1 [12]	0–1; higher score indicates better HRQoL
PROMIS® GH	Generic (HRQoL)	Previously used in NF1 PN [11]	0–100; higher score indicates better HRQoL
INF1-QOL	NF1-specific (HRQoL)	Validated for assessment of HRQoL in patients with NF1, with significant bivariate correlations reported with EQ-5D and physician rated clinical severity scores [12]	0–42; higher score indicates worse HRQoL
**Caregivers of paediatric patients**			
EQ-5D-5L	Generic (HRQoL)	Previously used to measure caregiver HRQoL [17, 18]	0–1; higher score indicates better HRQoL
CarerQoL	Caregiver-specific (care-related Qol)	Used in informal care research and economic evaluation; validated for assessment of care-related QoL in informal caregivers, with significant multivariate correlations identified with other measures such as EQ-5D [19–22]	0–100; higher score indicates better care-related QoL
WPAI:CG	Caregiver-specific (work productivity)	Validated for assessment of productivity loss in caregivers through evidence of strong, significant correlations with existing measures [23, 24]	0%–100%; higher score indicates greater impairment to work productivity

*Abbreviations*: *CarerQol* Care-Related Quality of Life, *HRQoL* health-related quality of life, *INF1-QOL* Impact of NF1 on Quality of Life, *NF1* neurofibromatosis type 1, *PN* plexiform neurofibroma(s), *PROMIS® GH* Patient-Reported Outcomes Measurement Information System® Global Health, *QoL* quality of life, *WPAI:CG* Work Productivity and Activity Impairment questionnaire, as adapted for caregiving

All measures were integrated into the digital survey. EuroQol and HealthMeasures provided approval of the digital versions of EQ-5D-5L and PROMIS® GH, respectively, for use in the survey.

### Statistical analysis

Incomplete survey responses were not excluded from the study. Implausible or numerically/clinically illogical entries were removed (e.g. where reported time since NF1 diagnosis was greater than age). For categorical variables, clinically similar categories with a low number of responses were merged (e.g. responses indicating PN location on the head or on the neck were merged into a single category, “head/neck”).

Descriptive statistics were generated for demographic and clinical characteristics, HRQoL outcomes and work productivity outcomes. EQ-5D-3L utility scores were estimated from EQ-5D-5L responses using the validated cross-walk by van Hout et al. [25, 26], in line with the preferences stated by the National Institute for Health and Care Excellence (NICE) at the time of study conception [27], and are hereafter referred to as EQ-5D utilities. PROMIS® GH physical and mental health scores (PHS and MHS, respectively) were calculated by summing the relevant items before transforming to a T-score [28]. Summary scores were calculated from INF1-QOL responses. Weighted sum scores were calculated from CarerQol responses based on public preferences for different caregiving situations [29]. Scores from WPAI:CG responses were calculated for absenteeism, presenteeism, productivity loss and regular activity productivity loss [23].

A set of exploratory univariable analyses was conducted by fitting simple linear regression models to investigate associations between patient characteristics (as explanatory variables) and HRQoL scores (as outcome variables). The resultant coefficient estimates from the regression models corresponded to the expected change in HRQoL score for every unit change in the patient characteristic. The corresponding 95% confidence intervals (CI) and p-values were reported. Statistical testing was conducted at the 5% significance level. The impact of the presence of symptoms on HRQoL was explored by treating symptoms as a combined variable (i.e. the presence of any symptom versus no symptoms). In order to explore associations between the presence of selected individual symptoms (versus the absence of the symptom) and HRQoL, post hoc exploratory univariable analyses were conducted using the same methods.

## Results

### HRQoL of adult patients

#### Demographic and clinical characteristics

Fifty-one eligible adult patients started the survey; 35/51 patients provided answers to all questions relating to their clinical and demographic characteristics. The numbers of patients who answered each specific question ranged from 33–49 and are shown in Additional file 1: Supplementary table 1.

Demographic and clinical characteristics are provided in Additional file 1: Supplementary table 1. Mean patient age was 37.5 years (standard deviation [SD]: 13.6; *n* = 48), 75.5% (37/49) of patients were female and mean body surface area (BSA; estimated surface area of a patient’s whole body based on height and weight) was 1.76 m^2^ (SD: 0.20; calculated using 30 response sets with plausible values for height and weight). The mean reported number of PN was 2.39 (SD: 2.64; *n* = 23; ten patients answered “don’t know” for this question). PN were most commonly located on the head (14/35 [40.0%]), back (10/35 [28.6%]) and extremities (arms: 9/35 [25.7%]; and legs: 9/35 [25.7%]). Most patients (31/35 [88.6%]) were currently experiencing at least one symptom, such as disfigurement (22/35 [62.9%]), pain (19/35 [54.3%]), bowel or bladder dysfunction (5/35 [14.3%]), vision loss (4/35 [11.4%]) and motor dysfunction (4/35 [11.4%]). Difficulty swallowing was reported by 22.9% (8/35) of patients. The majority (22/35 [62.9%]) of patients reported at least one comorbidity. Chronic itching was experienced by 9/35 (25.7%) patients. Partial PN resection was reported by 17/35 (48.6%) patients, and complete resection by 3/35 (8.57%) patients.

#### HRQoL scores

HRQoL results are presented in Table 2. Of the 51 eligible patients who started the survey, scores were estimated from the 35 patients who completed EQ-5D and PROMIS® GH, and from the 33 patients who completed INF1-QOL. The mean estimated EQ-5D utility was 0.65 (SD: 0.29; *n* = 35), substantially worse than the age- and sex-matched general population estimated mean of 0.89 (SD: 0.04). Substantial numbers of patients reported moderate to extreme pain/discomfort (14/35 [40%]) and moderate to extreme anxiety/depression (18/35 [51.4%]).

**Table 2:** HRQoL outcomes for eligible adult patients

Variable	Value (*N* = 51)
**EQ-5D-5L**^**a**^	
Number of responses, n	35
**Mobility**	
No problems, n (%)	21 (60.0)
Slight problems, n (%)	11 (31.4)
Moderate problems, n (%)	2 (5.71)
Severe problems, n (%)	1 (2.86)
Unable to walk about, n (%)	0
**Self-care**	
No problems, n (%)	28 (80.0)
Slight problems, n (%)	6 (17.1)
Moderate problems, n (%)	1 (2.86)
Severe problems, n (%)	0
Unable to wash/dress, n (%)	0
**Usual activities**	
No problems, n (%)	19 (54.3)
Slight problems, n (%)	9 (25.7)
Moderate problems, n (%)	6 (17.1)
Severe problems, n (%)	0
Unable to do, n (%)	1 (2.86)
**Pain/discomfort**	
No pain or discomfort, n (%)	11 (31.4)
Slight pain or discomfort, n (%)	10 (28.6)
Moderate pain or discomfort, n (%)	8 (22.9)
Severe pain or discomfort, n (%)	5 (14.3)
Extreme pain or discomfort, n (%)	1 (2.86)
**Anxiety/depression**	
Not anxious or depressed, n (%)	6 (17.1)
Slightly anxious or depressed, n (%)	11 (31.4)
Moderately anxious or depressed, n (%)	12 (34.3)
Severely anxious or depressed, n (%)	4 (11.4)
Extremely anxious or depressed, n (%)	2 (5.71)
**Utility score**^**a**^	
Mean (± SD)	0.65 (± 0.29)
Median	0.72
IQR	0.37
Minimum–maximum	-0.15–1.00
**PROMIS® GH**	
Number of estimable scores, n	35
**Physical health score**	
Mean (± SD)	43.6 (± 9.19)
Median	44.9
IQR	13.4
Minimum–maximum	26.7–61.9
**Mental health score**	
Mean (± SD)	41.7 (± 11.5)
Median	38.8
IQR	17.0
Minimum–maximum	25.1–67.7
**Estimated EQ-5D utility score (from PROMIS® GH)**	
Mean (± SD)	0.65 (± 0.11)
Median	0.65
IQR	0.14
Minimum–maximum	0.42–0.84
**Overall health**	
Excellent, n (%)	3 (8.57)
Very good, n (%)	5 (14.3)
Good, n (%)	16 (45.7)
Fair, n (%)	6 (17.1)
Poor, n (%)	5 (14.3)
**INF1-QOL**	**Value (*N* = 51)**
Number of estimable scores, n	33
**Total score**	
Mean (± SD)	11.03 (± 6.02)
Median	11.00
IQR	7.00
Minimum–maximum	1–26

*Abbreviations*: *HRQoL* health-related quality of life, *INF1-QOL* Impact of NF1 on Quality of Life, *IQR* interquartile range, *NF1* neurofibromatosis type 1, *PROMIS® GH* Patient-Reported Outcomes Measurement Information System® Global Health, *SD* standard deviation

^a^Responses from EQ-5D-5L were cross-walked to EQ-5D-3L utility scores using the validated mapping function by van Hout et al. (based on the UK 3L value set) [25, 26]. A higher EQ-5D utility and PROMIS® GH score, and a lower INF1-QOL score, indicate better HRQoL

In patients who completed the PROMIS® GH measure, mean PHS and MHS were 43.6 (SD: 9.2; *n* = 35) and 41.7 (SD: 11.5; *n* = 35), respectively, where the general population have a mean score of 50.0 (SD: 10.0). The mean score for patients who completed INF1-QOL was 11.03 (SD: 6.02; *n* = 33).

#### Associations between patient characteristics and HRQoL

**Fig. 1:**
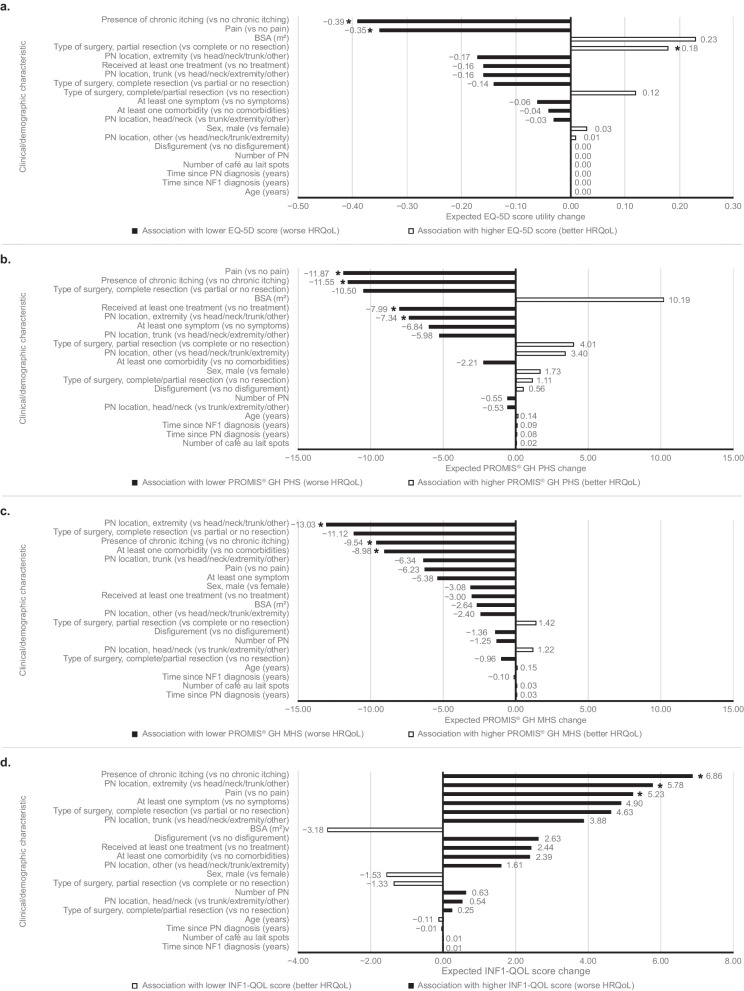
Tornado plots of univariable analyses of clinical or demographic characteristics of adult patients and HRQoL outcomes. **a** Associations with EQ-5D utility; **b** Associations with PROMIS GH® PHS; **c** Associations with PROMIS GH® MHS; **d** Associations with INF1-QOL score. * denotes statistically significant associations (*p* < 0.05). Results from exploratory simple linear regression models. Abbreviations: BSA: body surface area; HRQoL: health-related quality of life; INF1-QoL: Impact of NF1 on Quality of Life; MHS: mental health score; NF1: neurofibromatosis type 1; PHS: physical health score; PROMIS® GH: Patient-Reported Outcomes Measurement Information System® Global Health; PN: plexiform neurofibroma(s)

Results of the univariable analysis are presented in Fig. 1a–d (full results in Additional file 1: Supplementary table 2). PN location on arms and/or legs (bodily extremity) compared with any other PN location was associated with a worse PROMIS® GH PHS (*p* = 0.024) and MHS (*p* < 0.001) and worse INF1-QOL score (*p* = 0.011), but this result was not statically significant for EQ-5D (utility change: -0.37 [i.e. worse], *p* = 0.104). In addition, better HRQoL was observed with higher BSA but this trend was not statistically significant for any measure (expected EQ-5D utility change: + 0.23 per 1 m^2^ BSA increase; *p* = 0.341).

Presence of at least one comorbidity, versus no comorbidities, was significantly associated with worse PROMIS® GH MHS (*p* = 0.037); this result was not significant for EQ-5D (utility change: -0.04; *p* = 0.730). Worse HRQoL scores were observed with the presence of at least one symptom, versus no symptoms, but this result was not significant for any measure (EQ-5D utility change: -0.06; *p* = 0.778). Presence of chronic itching was associated with worse HRQoL; this result was significant for EQ-5D (utility change: -0.39; *p* < 0.001), PROMIS® GH physical health score (*p* = 0.001), PROMIS® GH MHS (*p* = 0.038) and INF1-QOL score (*p* = 0.004).

In the post hoc analysis, the presence of pain was found to be significantly associated with worse HRQoL compared with no pain for EQ-5D (utility change: -0.35; *p* < 0.001), PROMIS® GH PHS (*p* < 0.001) and INF1-QOL score (*p* = 0.01). The presence of disfigurement, compared with no disfigurement, was not found to be associated with HRQoL (EQ-5D utility change: 0.00; *p* = 0.979).

### HRQoL and work productivity of caregivers

#### Demographic and clinical characteristics

Eight caregivers completed the survey. Demographic and clinical characteristics are provided in Additional file 1: Supplementary table 3. Mean age was 45.0 years (SD: 9.24; *n* = 8) and 75.0% were female. Most caregivers (7/8 [87.5%]) were the primary caregiver of one child with NF1 PN. Half of the caregivers were also employed full-time and one caregiver (1/8 [12.5%]) reported being unable to work due to caring for someone with NF1 PN. Seven caregivers confirmed that neither they, nor the other parent of the child they care for, had a diagnosis of NF1 and six confirmed no diagnosis of PN amongst either parent; the remaining responses were “prefer not to say” or “don’t know”. Presence of at least one comorbidity was reported by 5/8 (62.5%) caregivers, including anxiety and depression (2/8 [25.0%] each).

#### HRQoL and work productivity scores

HRQoL and work productivity results (overall and by domain) are presented in Table 3. All eight caregivers completed EQ-5D-5L, CarerQol and WPAI:CG. However, each question of WPAI:CG was only applicable to certain caregivers based on their employment status.

**Table 3:** HRQoL and work productivity outcomes for eligible caregivers

Variable	Value
**EQ-5D-5L**^**a**^	**(*N* = 9)**
Number of responses, n	8
**Mobility**	
No problems, n (%)	5 (62.5)
Slight problems, n (%)	2 (25.0)
Moderate problems, n (%)	1 (12.5)
Severe problems, n (%)	0
Unable to walk about, n (%)	0
**Self-care**	
No problems, n (%)	7 (87.5)
Slight problems, n (%)	1 (12.5)
Moderate problems, n (%)	0
Severe problems, n (%)	0
Unable to wash/dress, n (%)	0
**Usual activities**	
No problems, n (%)	5 (62.5)
Slight problems, n (%)	2 (25.0)
Moderate problems, n (%)	0
Severe problems, n (%)	0
Unable to do, n (%)	1 (12.5)
**Pain/discomfort**	
No pain or discomfort, n (%)	3 (37.5)
Slight pain or discomfort, n (%)	1 (12.5)
Moderate pain or discomfort, n (%)	4 (50.0)
Severe pain or discomfort, n (%)	0
Extreme pain or discomfort, n (%)	0
**Anxiety/depression**	
Not anxious or depressed, n (%)	4 (50.0)
Slightly anxious or depressed, n (%)	2 (25.0)
Moderately anxious or depressed, n (%)	1 (12.5)
Severely anxious or depressed, n (%)	1 (12.5)
Extremely anxious or depressed, n (%)	0
**Utility score**^**a**^	
Mean (± SD)	0.72 (± 0.24)
Median	0.74
IQR	0.28
Minimum–maximum	0.31–1.00
**CarerQol**	**Value**
Number of responses, n	8
**Utility score**	
Mean (± SD)	69.3 (± 13.9)
Median	66.3
IQR	22.1
Minimum–maximum	51.8–91.4
**Happiness score**	
Mean (± SD)	5.63 (± 1.77)
Median	6.50
IQR	3.00
Minimum–maximum	3.00–7.00
**WPAI:CG**	**Value (*N* = 9)**
**Absenteeism**	
Number of estimable scores, n	6
Mean (± SD), %	24.5 (± 37.9)
Median, %	13.5
IQR, %	20.0
Minimum–maximum, %	0.00–100
**Presenteeism**	
Number of estimable scores, n	5
Mean (± SD), %	40.0 (± 39.4)
Median, %	40.0
IQR, %	40.0
Minimum–maximum, %	0.00–100
**Productivity loss**	
Number of estimable scores, n	5
Mean (± SD), %	42.9 (± 40.1)
Median, %	46.2
IQR, %	48.3
Minimum–maximum, %	0.00–100
**Regular activity productivity loss**	
Number of estimable scores, n	8
Mean (± SD), %	36.3 (± 31.6)
Median, %	30.0
IQR, %	35.0
Minimum–maximum, %	0.00–100

*Abbreviations*: *CarerQol* Care-Related Quality of Life, *HRQoL* health-related quality of life, *IQR* interquartile range, *NF1* neurofibromatosis type 1, *PN* plexiform neurofibroma(s), *SD* standard deviation, *WPAI:CG* Work Productivity and Activity Impairment questionnaire, as adapted for caregiving

^a^Responses from EQ-5D-5L were cross-walked to EQ-5D-3L utility scores using the validated mapping function by van Hout et al. (based on the UK 3L value set) [25, 26]. COVID-19 refers to the coronavirus pandemic. A higher EQ-5D utility and CarerQol utility score indicate better HRQoL. A higher percentage score in WPAI:CG represents a greater impairment to work productivity

The mean EQ-5D utility was 0.72 (SD: 0.24), substantially worse than the age- and sex-matched general population estimated mean of 0.88 (SD: 0.03). Moderate pain/discomfort was reported by 4/8 (50.0%) caregivers, and slight to severe problems with anxiety/depression were reported by 4/8 (50.0%) caregivers with EQ-5D. The CarerQol overall utility score was 69.3 (SD: 13.9).

In the WPAI:CG, almost a quarter of worktime was missed due to caregiving (mean absenteeism: 24.5% [SD: 37.9%; *n* = 6]) and productivity whilst at work was impaired by 40.0% (mean presenteeism; SD: 39.4%; *n* = 5). This led to a mean productivity loss of 42.9% (SD: 40.1%; *n* = 5). Mean regular activity productivity loss (i.e. extent to which caregiving impaired productivity while carrying out daily activities) was 36.3% (SD: 31.6%; *n* = 8).

## Discussion

### Adult patients experience a substantial impact of NF1 PN on HRQoL

To our knowledge, this study is the first to use validated measures to explore HRQoL and estimate health utilities directly from adult patients with NF1 PN. Results across both generic (EQ-5D-5L and PROMIS® GH) and disease-specific (INF1-QOL) measures suggest that NF1 PN has a considerable impact on patients across a broad range of HRQoL domains (including physical, social and emotional heath and daily activities). In accordance with previous studies [12, 13], our study highlights the impact of NF1 PN on anxiety/depression and pain/discomfort, with substantial proportions of patients reporting moderate to extreme problems in these domains.

The majority of patients reported a range of comorbidities; the presence of comorbidities was significantly associated with a poorer PROMIS® GH MHS, suggesting that they may add to the impact on patients’ mood, ability to think and social activities. The lack of statistically significant association between the presence of NF1 PN symptoms and worse HRQoL may be an artefact of a small number (2/35 [5.7%]) of patients reporting no symptoms. However, of note, the presence of pain (reported by 19/35 [54.3%] patients) was significantly associated with worse HRQoL scores. This reflected the results of a previous study of adult patients with NF1 [13], in which over 60% reported pain and a significant association was found between increased pain interference and decreased HRQoL.

PN location at extremities (arms/legs), compared with on the head/neck, trunk or other location, was significantly associated with poorer HRQoL. This potentially highlights interference with daily activities due to PN located at extremities, and has previously been reported in sarcoma [30].

Negative experiences with disfigurement were noted in patients’ written responses to INF1-QOL in this study (Additional file 1: Supplementary table 4), and in interviews in a previous study [9]. However, the presence of disfigurement as a symptom of NF1 PN was found to have no significant association with HRQoL in our study. This result may arise from the small sample size and use of generic HRQoL measures, resulting in a lack of powering to detect a significant result. Severity of disfigurement was not considered in this study.

Previous literature has also demonstrated that patients with NF1 have substantially poorer HRQoL than the general population. The mean EQ-5D utility estimated for adult patients in this study (0.65) was substantially worse than the age- and sex-matched general population estimated mean (0.89) demonstrating the impact of NF1 PN on HRQoL of patients. Additionally, the EQ-5D utility and INF1-QOL score (11.03) in patients with NF1 PN in this study indicated worse HRQoL than in previous studies of adult patients with NF1 regardless of the presence of PN (EQ-5D: 0.73 [39% of patients had PN] [13]; INF1-QOL: 8.64 [12]). This suggests that the impact of NF1 PN on HRQoL is more substantial than the previously reported impact of NF1.

By demonstrating the impact of NF1 PN on the HRQoL of patients, the results of this study highlight the unmet need for an improvement to established clinical management of NF1 PN, in particular to address the impacts of PN located at extremities, comorbidities and pain on HRQoL, thereby reducing the burden experienced by patients.

### NF1 PN has a substantial, negative impact on caregiver HRQoL and work productivity

In accordance with previous studies [15, 16], this study demonstrates the impact of caregiving for patients with NF1 PN on HRQoL and work productivity. Although half of NF1 cases are inherited, no caregivers in this study confirmed having NF1 themselves, so results are unlikely to capture the additional burden experienced by caregivers who have NF1 themselves.

Results across generic (EQ-5D-5L) and care-related (CarerQol, WPAI:CG) measures suggest that caring for patients with NF1 PN impairs HRQoL, finances and productivity. Importantly, the mean EQ-5D utility estimated for caregivers in this study (0.72) indicated worse HRQoL than the age- and sex-matched general population estimated mean (0.88). Furthermore, the CarerQol utility score (69.3), indicated worse care-related QoL than that reported in a previous study of informal caregivers of patients with a range of diseases (76.0) [31], highlighting the negative impact of NF1 PN on care-related QoL. In this study, the CarerQol utility score was worse than the EQ-5D utility value. Whilst CarerQol scores have previously been shown to be moderately correlated with EQ-5D scores in convergent validity studies [21, 22], the focus of CarerQol on specific caregiving issues rather than general health may make it more sensitive than EQ-5D to care-related QoL impacts. It is also worth noting that EQ-5D may have captured the HRQoL impacts of factors unrelated to caring for a patient with NF1 PN, e.g. of comorbidities which were reported by 5/8 (62.5%) caregivers.

Two out of eight (25%) caregivers each reported experiencing anxiety or depression in this study. In a different study of caregivers of patients with NF1 PN in the United States of America (USA), 48.4% and 34.7% of caregivers reported experiencing anxiety and depression, respectively, using the Zarit Burden Interview [16].

Amongst the caregivers who were also employed part- or full-time, results of the WPAI:CG questionnaire demonstrated that a quarter of worktime was lost due to caregiving (absenteeism), and productivity whilst at work (presenteeism) was also decreased by almost half, resulting in a mean work productivity loss of 42.9%. The previously mentioned USA study of caregivers of patients with NF1 PN reported a large work productivity loss of 22.3% with the WPAI:CG [16]; this productivity loss may be smaller than in the current study due to differing characteristics of the caregivers or children they care for, or sociocultural differences between the USA and UK. The productivity losses reported in both studies are much greater than the 11.7% reported by a UK general population study in 2017 with 31,950 employees, using the WPAI [32].

Limitations of this study include the low participant numbers, especially in the caregiver survey, possibly leading to uncertainty and lack of powering (as evidenced by the wide CIs). Despite heterogeneity in the clinical manifestations of NF1 PN and the low participant numbers, the similar mean and median health utilities in each population indicate that skewness is unlikely. Given the nature of this rare disease, qualitative interviews or mixed methods may improve results obtained in the future. Collection of data through an online, survey-based approach may have resulted in voluntary response bias and sampling bias. Advertising the survey solely in an electronic format enabled anonymity, however, likely prevented a minority of people from being able to participate in the survey. This study was cross-sectional and exploratory. Hence, it was not possible to infer causal relationships, and results from significance testing and comparisons with existing literature must be interpreted with care.

## Conclusions

This study captures the real-world, lived experience of adult patients with NF1 PN receiving established care, and of caregivers of paediatric patients. Patients with NF1 PN experience pain and heterogeneous comorbidities, affecting both mental and physical health. This HRQoL impact also extends to caregivers, who additionally reported experiencing substantial productivity loss as a result of caring. Health utilities were worse for adult patients and caregivers of paediatric patients than the general population, with pain/discomfort and anxiety/depression affected for both groups. This study could be supplemented by further research using qualitative data to better understand the patient and caregiver experience in NF1 PN, and would benefit from a larger sample size and geographical scope. Additionally, as all analyses in this study were exploratory in nature, further research could pre-specify confirmatory analyses to allow inference of causal relationships. This study highlights an unmet need for better management of NF1 PN to reduce the burden experienced by patients and caregivers.

### Supplementary Information

**Additional file 1.**

## Data Availability

All data generated or analysed during this study are included in this published article and its supplementary information files.

## Abbreviations

BSA: Body surface area
CarerQol: Care-Related Quality of Life
CI: Confidence interval
HRQoL: Health-related quality of life
INF1-QOL: Impact of NF1 on Quality of Life
IRAS: Integrated Research Application System
MEK: Mitogen-activated protein kinase kinase
MHS: Mental health score
NF1: Neurofibromatosis type 1
NICE: National Institute for Health and Care Excellence
NTUK: Nerve Tumours UK
PHS: Physical health score
PN: Plexiform neurofibroma(s)
PROMIS® GH: Patient-Reported Outcomes Measurement Information System® Global Health
QoL: Quality of life
SD: Standard deviation
UK: United Kingdom
USA: United States of America
WPAI: Work Productivity and Activity Impairment Questionnaire
WPAI:CG: WPAI questionnaire, as adapted for caregiving
