# Supplementary material for: Impact of neurofibromatosis type 1 with plexiform neurofibromas on the health-related quality of life and work productivity of adult patients and caregivers in the UK: a cross-sectional survey
Source: BMC Neurol. 2023 Nov 23;23:419. doi: 10.1186/s12883-023-03429-7 (PMC10666383; doi:10.1186/s12883-023-03429-7)
Supplement: Supplementary file 1 — Additional file 1. [file 12883_2023_3429_MOESM1_ESM.pdf]

Additional file 1

Impact of neurofibromatosis type 1 with plexiform neurofibromas on the health-related quality of life of adult patients and caregivers in the UK: a cross-sectional survey

Hyun Kyoo Yoo,[1] Alex Porteous, Alvin Ng, Keval Haria, Annabel Griffiths, Andrew Lloyd, Xiaoqin Yang, Gbenga Kazeem, Volkan Barut

Correspondence to:

Hyun Kyoo Yoo[1]

[1]Alexion, AstraZeneca Rare Disease, Cambridge, United Kingdom

HyunKyoo.Yoo@alexion.com

ORCID ID: 0000-0001-7237-7358

Supplementary table 1 Demographic and clinical characteristics of overall adult population

Variable	Value (N=51)
**Age (years)**	
Number of responses, n	49
Number of indicated values, n (%)	48 (98.0)
Mean (±SD)	37.5 (±13.6)
Median	34.0
IQR	17.5
Minimum–maximum	19.0–71.0
Prefer not to say, n (%)	1 (2.04)
Don't know, n (%)	0
Missing, n	2
**Sex**	
Number of responses, n	49
Male, n (%)	12 (24.5)
Female, n (%)	37 (75.5)
Prefer not to say, n (%)	0
Don't know, n (%)	0
Missing, n	2
**Height (cm)**	
Number of responses, n	47
Number of indicated values, n (%)	37 (78.7)
Mean (±SD)	162 (±8.97)
Median	162
IQR	13.0
Minimum–maximum	147–187
Prefer not to say, n (%)	4 (8.51)
Don't know, n (%)	6 (12.8)
Missing, n	4[a]
**Weight (kg)**	
Number of responses, n	47
Number of indicated values, n (%)	30 (63.8)
Mean (±SD)	71.0 (±17.1)
Median	66.5
IQR	20.9
Minimum–maximum	51.0–114
Prefer not to say, n (%)	5 (10.6)
Don't know, n (%)	12 (25.5)
Missing, n	4
**BMI (kg/m^2)**	
Number of estimable values, n	30
Mean (±SD)	26.8 (±6.38)
Median	25.5
IQR	4.58
Minimum–maximum	19.2–45.1
Inestimable, n	21[a]
**BSA (m^2)**	
Number of estimable values, n	30
Mean (±SD)	1.76 (±0.20)

Median	1.67
IQR	0.29
Minimum–maximum	1.49–2.25
Inestimable, n	21[a]
**Presence of comorbidities**	
Number of responses, n	35
Headaches, n (%)	14 (40.0)
Attention-deficit disorder, n (%)	2 (5.71)
Autism, n (%)	2 (5.71)
Epilepsy, n (%)	1 (2.86)
Hypertension, n (%)	3 (8.57)
Congenital heart disease, n (%)	1 (2.86)
Vasculopathy, n (%)	0
Other, n (%)	9 (25.7)
None, n (%)	11 (31.4)
Prefer not to say, n (%)	0
Don't know, n (%)	0
Unclear, n (%)	2 (5.71)
Missing, n	16
**Time since NF1 diagnosis (years)**	
Number of responses, n	35
Number of indicated values, n (%)	34 (97.1)
Mean (±SD)	26.3 (±13.2)
Median	26.5
IQR	16.0
Minimum–maximum	5.00–71.0
Prefer not to say, n (%)	1 (2.86)
Don't know, n (%)	0
Unclear, n (%)	0
Missing, n	16
**Time since PN diagnosis (years)**	
Number of responses, n	35
Number of indicated values, n (%)	21 (60.0)
Mean (±SD)	22.9 (±14.7)
Median	22.0
IQR	19.0
Minimum–maximum	3.00–65.0
Prefer not to say, n (%)	2 (5.71)
Don't know, n (%)	12 (34.3)
Unclear, n (%)	0
Missing, n	16
**Number of café-au-lait spots**	
Number of responses, n	35
Number of indicated values, n (%)	16 (45.7)
Mean (±SD)	18.9 (±24.9)
Median	10.0
IQR	24.5
Minimum–maximum	0.00–100
Prefer not to say, n (%)	2 (5.71)
Don't know, n (%)	17 (48.6)
Missing, n	16

**Number of PN**	
Number of responses, n	33
Number of indicated values, n (%)	23 (69.7)
Mean (±SD)	2.39 (±2.64)
Median	1.00
IQR	1.00
Minimum–maximum	1.00–10.0
Prefer not to say, n (%)	0
Don't know, n (%)	10 (30.3)
Missing, n	18[b]
**Body parts with PN**	
Number of responses, n	35
Head, n (%)	14 (40.0)
Neck, n (%)	8 (22.9)
Chest, n (%)	6 (17.1)
Arms, n (%)	9 (25.7)
Legs, n (%)	9 (25.7)
Abdomen, n (%)	4 (11.4)
Spine, n (%)	5 (14.3)
Back, n (%)	10 (28.6)
Other, n (%)	12 (34.3)
Prefer not to say, n (%)	1 (2.86)
Don't know, n (%)	3 (8.57)
Unclear, n (%)	0
Missing, n	16
**Symptoms**	
Number of responses, n	35
Disfigurement, n (%)	22 (62.9)
Pain, n (%)	19 (54.3)
Motor dysfunction, n (%)	4 (11.4)
Airway obstruction, n (%)	0
Vision loss, n (%)	4 (11.4)
Bowel or bladder dysfunction, n (%)	5 (14.3)
Other, n (%)	2 (5.71)
None, n (%)	2 (5.71)
Prefer not to say, n (%)	1 (2.86)
Don't know, n (%)	0
Unclear, n (%)	1 (2.86)
Missing, n	16
**Presence and treatment of chronic itching**	
Number of responses, n	35
Chronic itching and treated, n (%)	1 (2.86)
Chronic itching and not treated, n (%)	8 (22.9)
No chronic itching, n (%)	24 (68.6)
Prefer not to say, n (%)	0
Don't know, n (%)	2 (5.71)
Missing, n	16
**Treatments received**	
Number of responses, n	35
Pain relievers, n (%)	11 (31.4)
Oncology medications, n (%)	1 (2.86)

Radiation, n (%)	1 (2.86)
Surgery, n (%)	17 (48.6)
Other, n (%)	2 (5.71)
None, n (%)	11 (31.4)
Prefer not to say, n (%)	0
Don't know, n (%)	0
Unclear, n (%)	3 (8.57)
Missing, n	16
**Surgeries received**	
Number of responses, n	35
Complete resection, n (%)	3 (8.57)
Partial resection, n (%)	17 (48.6)
None, n (%)	14 (40.0)
Prefer not to say, n (%)	0
Don't know, n (%)	2 (5.71)
Unclear, n (%)	0
Missing, n	16
**Difficulty swallowing**	
Number of responses, n	35
Yes, n (%)	8 (22.9)
No, n (%)	27 (77.1)
Prefer not to say, n (%)	0
Don't know, n (%)	0
Missing, n	16

Footnotes: [a]Two implausible entries for height were removed, and thus did not inform BSA and BMI calculations. Plausible values for both height and weight were required to calculate BSA and BMI. [b]Two entries deemed to be implausible were removed.

Abbreviations: BMI: body mass index; BSA: body surface area; IQR: interquartile range; NF1: neurofibromatosis type 1; PN: plexiform neurofibroma(s); SD: standard deviation.

Supplementary Table 2 Results of univariable analyses in the adult population

Variable/Group	EQ-5D utility[a]				PROMIS GH® PHS				PROMIS GH® MHS				INF1-QOL			
	n	Coefficient	95% CI	p-value	n	Coefficient	95% CI	p-value	n	Coefficient	95% CI	p-value	n	Coefficient	95% CI	p-value
Age (years)	34	0.00	0.00, 0.01	0.206	34	0.14	-0.08, 0.37	0.198	34	0.15	-0.12, 0.42	0.268	32	-0.11	-0.25, 0.04	0.161
BSA (m²)	21	0.23	-0.26, 0.72	0.341	21	10.19	-11.27, 31.65	0.333	21	-2.64	-27.99, 22.71	0.830	19	-3.18	-12.11, 5.76	0.464
Sex	35				35				35				33			
Female	26		Reference		26		Reference		26		Reference		25		Reference	
Male	9	0.03	-0.20, 0.26	0.791	9	1.73	-5.58, 9.04	0.633	9	-3.08	-12.22, 6.06	0.498	8	-1.53	-6.56, 3.51	0.541
Time since NF1 diagnosis	34	0.00	0.00, 0.01	0.209	34	0.09	-0.15, 0.33	0.447	34	-0.10	-0.39, 0.19	0.503	32	0.01	-0.15, 0.17	0.913
Time since PN diagnosis	21	0.00	-0.01, 0.01	0.404	21	0.08	-0.21, 0.38	0.561	21	0.03	-0.31, 0.38	0.852	20	-0.01	-0.19, 0.18	0.950
Number of café-au-lait spots	16	0.00	-0.01, 0.01	0.846	16	0.02	-0.21, 0.24	0.880	16	0.03	-0.22, 0.28	0.798	16	0.01	-0.13, 0.15	0.875
Number of PN	23	0.00	-0.05, 0.05	0.880	23	-0.55	-2.20, 1.10	0.495	23	-1.25	-3.16, 0.66	0.187	22	0.63	-0.46, 1.73	0.243
Comorbidities	33				33				33				31			
None	11		Reference		11		Reference		11		Reference		11		Reference	
At least one comorbidity	22	-0.04	-0.26, 0.18	0.730	22	-2.21	-9.19, 4.77	0.523	**22**	**-8.98**	**-17.41, -0.55**	**0.037**	20	2.39	-2.17, 6.95	0.292
PN location[b]	31				31				31				29			
Head/neck	16	-0.03	-0.24, 0.18	0.778	16	-0.53	-7.35, 6.28	0.874	16	1.22	-6.97, 9.41	0.762	15	0.54	-4.19, 5.28	0.816
Trunk	15	-0.16	-0.36, 0.04	0.113	15	-5.98	-12.40, 0.45	0.067	15	-6.34	-14.18, 1.50	0.109	14	3.88	-0.61, 8.36	0.088
Extremity	13	-0.17	-0.37, 0.04	0.104	**13**	**-7.34**	**-13.65, -1.03**	**0.024**	**13**	**-13.03**	**-19.70, -6.35**	**<0.001**	**11**	**5.78**	**1.47, 10.10**	**0.011**
Other	12	0.01	-0.21, 0.23	0.935	12	3.40	-3.48, 10.27	0.321	12	-2.40	-10.76, 5.96	0.562	12	1.61	-3.15, 6.38	0.494
Symptom	33				33				33				31			
None	2		Reference		2		Reference		2		Reference		2		Reference	
At least one symptom	31	-0.06	-0.49, 0.37	0.778	31	-6.84	-20.35, 6.68	0.310	31	-5.38	-20.08, 9.33	0.462	29	4.90	-3.68, 13.48	0.253
Presence of chronic itching	33				33				33				31			
No	24		Reference		24		Reference		24		Reference		23		Reference	
Yes	**9**	**-0.39**	**-0.58, -0.20**	**<0.001**	**9**	**-11.55**	**-17.86, -5.25**	**0.001**	**9**	**-9.54**	**-18.50, -0.58**	**0.038**	**8**	**6.86**	**2.31, 11.41**	**0.004**
Treatment	32				32				32				30			

None	11		Reference		11		Reference		11		Reference		11		Reference	
At least one treatment	21	-0.16	-0.35, 0.04	0.119	**21**	**-7.99**	**-14.59, -1.39**	**0.019**	21	-3.00	-12.14, 6.14	0.508	19	2.44	-2.17, 7.05	0.287
Type of surgery[b]	33				33				33				31			
Complete resection	3	-0.14	-0.46, 0.17	0.370	3	-10.50	-21.10, 0.09	0.052	3	-11.12	-25.09, 2.85	0.115	3	4.63	-2.47, 11.73	0.193
Partial resection	**17**	**0.18**	**0.00, 0.35**	**0.046**	17	4.01	-2.31, 10.32	0.205	17	1.42	-6.94, 9.77	0.732	17	-1.33	-5.65, 2.99	0.533
**Post hoc analyses**																
Symptom: disfigurement	33				33				33				31			
No	11		Reference		11		Reference		11		Reference		10		Reference	
Yes	22	0.00	-0.22, 0.22	0.98	22	0.56	-6.39, 7.52	0.870	22	-1.36	-8.86, 6.13	0.713	21	2.63	-1.88, 7.13	0.242
Symptom: pain	33				33				33				31			
No	14		Reference		14		Reference		14		Reference		14		Reference	
Yes	**19**	**-0.35**	**-0.52, -0.19**	**<0.001**	**19**	**-11.87**	**-16.88, -6.85**	**<0.001**	19	-6.23	-13.02, 0.56	0.071	**17**	**5.23**	**1.37, 9.08**	**0.010**
Surgery	33				33				33				31			
None	14		Reference		14		Reference		14		Reference		12		Reference	
Complete/partial resection	19	0.12	0.00, 0.00	0.00	19	1.11	-5.44, 7.65	0.733	19	-0.96	-9.41, 7.50	0.819	19	0.25	-4.19, 4.69	0.911

Footnotes: [a]Responses from EQ-5D-5L were cross-walked to EQ-5D-3L utility scores using the validated mapping function by van Hout et al. (based on the UK 3L value set) [1, 2]. [b]Reference group being none or any other of the listed groups; results should be interpreted with caution given the non-mutually exclusivity of the groups. Bold text denotes statistically significant associations ($p<0.05$). Results from exploratory simple linear regression models. A higher EQ-5D utility and PROMIS® GH score, and a lower INF1-QOL score, indicate better HRQoL.

Abbreviations: BSA: body surface area; CI: confidence interval; HRQoL: health-related quality of life; INF1-QoL: Impact of NF1 on Quality of Life; MHS: mental health score; NF1: neurofibromatosis type 1; PHS: physical health score; PROMIS® GH: Patient-Reported Outcomes Measurement Information System® Global Health; PN: plexiform neurofibroma(s).

Supplementary table 3 Characteristics of overall caregiver population

Variable	Value (N=9)
**Primary caregiver**	
Number of responses, n	8
Yes, of 1 person with NF1 PN, n (%)	7 (87.5)
Yes, of ≥1 person with NF1 PN, n (%)	0
No, n (%)	1 (12.5)
Prefer not to say, n (%)	0
Don't know, n (%)	0
Missing, n	1
**Number of caregivers of the person with NF1 PN**	
Number of responses, n	8
Number of indicated values, n (%)	4 (50.0)
Mean (±SD)	1.00 (±0)
Median	1.00
IQR	0
Minimum–maximum	1.00–1.00
Prefer not to say, n (%)	4 (50.0)
Don't know, n (%)	0
Unclear, n (%)	0
Missing, n	1
**Age (years)**	
Number of responses, n	8
Number of indicated values, n (%)	7 (87.5)
Mean (±SD)	45.0 (±9.24)
Median	41.0
IQR	16.0
Minimum–maximum	32.0–55.0
Prefer not to say, n (%)	1 (12.50)
Don't know, n (%)	0
Missing, n	1
**Sex**	
Number of responses, n	8
Male, n (%)	2 (25.0)
Female, n (%)	6 (75.0)
Prefer not to say, n (%)	0
Don't know, n (%)	0
Missing, n	1
**Ethnicity**	
Number of responses, n	8
White, n (%)	8 (100.00)
Asian, n (%)	0
Black, n (%)	0
Prefer not to say, n (%)	0
Don't know, n (%)	0
Other, n (%)	0
Missing, n	1
**Caregiver's and other parent's diagnosis of NF1**	
Number of responses, n	8

Both diagnosed, n (%)	0
Only caregiver diagnosed, n (%)	0
Only other parent diagnosed, n (%)	0
Neither diagnosed, n (%)	7 (87.5)
Prefer not to say, n (%)	1 (12.5)
Don’t know, n (%)	0
Missing, n	1
**Caregiver’s and other parent’s diagnosis of PN**	
Number of responses, n	8
Both diagnosed, n (%)	0
Only caregiver diagnosed, n (%)	0
Only other parent diagnosed, n (%)	0
Neither diagnosed, n (%)	6 (75.0)
Prefer not to say, n (%)	1 (12.5)
Don’t know, n (%)	1 (12.5)
Missing, n	1
**Presence of comorbidities**	
Number of responses, n	8
Anxiety, n (%)	2 (25.0)
Cancer, n (%)	0
Depression, n (%)	2 (25.0)
Diabetes, n (%)	1 (12.5)
Obesity, n (%)	1 (12.5)
Other, n (%)	1 (12.5)
None, n (%)	0
Prefer not to say, n (%)	0
Don't know, n (%)	0
Unclear, n (%)	3 (37.5)
Missing, n	1
**Marital status**	
Number of responses, n	8
Married or domestic partnership, n (%)	7 (87.5)
Divorced/separated, n (%)	0
Single, never married, n (%)	0
Widowed, n (%)	1 (12.5)
None of the above, n (%)	0
Prefer not to say, n (%)	0
Don't know, n (%)	0
Missing, n	1
**Current employment**	
Number of responses, n	8
Employed/self-employed, full-time, n (%)	4 (50.0)
Employed/self-employed, part-time (due to caring for someone with NF1 PN), n (%)	0
Employed/self-employed, part-time (due to another reason), n (%)	1 (12.5)
Homemaker, n (%)	1 (12.5)
Unemployed, seeking work, n (%)	0
Unable to work due to health issues, n (%)	0
Unable to work due to caring for someone with NF1 PN, n (%)	1 (12.5)
Retired, n (%)	0
Student, n (%)	0

Other, n (%)	0
Prefer not to say, n (%)	1 (12.5)
Don't know, n (%)	0
Unclear, n (%)	0
Missing, n	1
**Impact of COVID-19 on employment status**	
Number of responses, n	8
No impact, n (%)	4 (50.0)
Pay and/or hours were reduced, n (%)	0
Lost job, n (%)	0
Work increased, n (%)	2 (25.0)
Not applicable, n (%)	1 (12.5)
Prefer not to say, n (%)	1 (12.5)
Don't know, n (%)	0
Missing, n	1

Footnotes: COVID-19 refers to the coronavirus pandemic.
Abbreviations: IQR: interquartile range; NF1: neurofibromatosis type 1; PN: plexiform neurofibroma(s); SD: standard deviation.

Supplementary Table 4 Written responses to INF1-QOL

"DIID NOT HAVE A FAMILY   CRUELNESS OF PEOPLE AS CHILD AND EVEN AS AN ADULT MAKING COMENTS ABOUT SKIN MARKINGS. PROBABLY NEVER MET MY FULL POTENTIAL AS NOR DIAGNOSED WHILST YOUNGER"
"I hate my skin, I hate the way people stare at me, I fully cover up in the summer and detest trying on clothes in changing rooms because they are so bright and highlights just how awful my skin actually is. I cannot have any of my tumours removed on the NHS because it's classed as plastic surgery, I've been told to go private but cannot afford to do so"
"As I have some learning disabilities my education was affected and I was limited in my employment choices. This has effect on how much I could earn. I only have a few friends and my social life is very limited. As I have got older more physical problems have developed, some very serious, and this makes me anxious about my future health."
"It's a constant sorce of anxiety."
"I was persistently led to believe as a child that I must never have children. While I came to rejectthis premise cognitively it nevertheless severely impacted my ability to make romantic and sexual relationships. Repeated hospitalisation as a child for surgery to tibial pseudoarthrosis also made an impact on my childhood and teenage relationships"
"Due to my plexiform being on my head and face it has grown my ear if I plug in head phones and squeeze it in I can hear fine. But it is much like a plug so creates hard to hear. And as a result unless I literally mop it out it is a bit of a bio hazard. I have had a bunch of ear infections especially if I swim a lot it tends to smell quite bad too."

Footnotes: Transferred directly from survey responses.

References

1. van Hout B, Janssen MF, Feng YS, Kohlmann T, Busschbach J, Golicki D, et al. Interim scoring for the EQ-5D-5L: mapping the EQ-5D-5L to EQ-5D-3L value sets. Value Health. 2012;15(5):708-15.

2. EQ-5D-5L | Valuation | Crosswalk Index Value Calculator. https://euroqol.org/eq-5d-instruments/eq-5d-5l-about/valuation-standard-value-sets/crosswalk-index-value-calculator/. Accessed 4 June 2021.
